# Effect of pH on recovery efficiency and anthocyanin composition of *Clitoria ternatea* extracts with application as lipid antioxidants in food preservation

**DOI:** 10.1002/fsn3.71786

**Published:** 2026-04-27

**Authors:** Kha Duyen Nguyen, Thi Thu Tra Tran, Ngọc Anh Le, Thi Anh Dao Dong

**Affiliations:** ^1^ Dept. Food Technology, Faculty of Chemical Engineering Ho Chi Minh City University of Technology (HCMUT), VNU HCM Ho Chi Minh city Vietnam

**Keywords:** anthocyanin, *Clitoria ternatea* ( *C. ternatea* ), DPPH antioxidant activity, LC‐HRMS, pH, polyphenol

## Abstract

This study aimed to examine the effect of pH on extraction efficiency, bioactive compounds, and potential food applications of anthocyanin‐rich extracts from *C. ternatea* flowers. Extractions were carried out under controlled conditions across a pH range of 1 to 13. The results indicated that total anthocyanin content (TAC) peaked at pH 3.0, while total polyphenol content (TPC) and DPPH antioxidant activity peaked at pH 7.0. These two pH conditions were further characterized using UV–Vis spectroscopy, scanning electron microscopy (SEM), and liquid chromatography–high‐resolution mass spectrometry (LC‐HRMS) to evaluate differences in absorbance spectra, cellular structures, and anthocyanin composition. LC‐HRMS analysis revealed a complex profile of anthocyanins and flavonoids, including Quercetin 3‐[2G]‐rhamnosylrutinoside, Delphinidin‐3‐(cis‐p‐coumaroyl‐glucoside), Kaempferol 3‐(6‐p‐coumaroyl)‐rutinoside, Kaempferol derivative, Cyanidin‐3‐(p‐coumaroyl)glucose, Kaempferol‐rhamnosyl‐malonyl‐glucoside, and Aglycoside cyanidin which were exclusively identified at the flower's natural pH (6.8–7.0). The observed compositional differences are associated with pH‐dependent structural transitions of anthocyanins from flavylium cations under acidic conditions to quinoidal base forms at near‐neutral pH. These findings provide insight into pH‐dependent anthocyanin behavior and offer guidance for optimizing extraction conditions for specific food applications. Anthocyanin and polyphenol extracts at pH 7 were tested for lipid oxidation in gummy candies. The results demonstrate the potential application of butterfly pea anthocyanins as functional ingredients in food systems, particularly as natural colorants with antioxidant functionality. Overall, this study provides a mechanistic understanding of pH‐dependent anthocyanin behavior and supports the use of neutral‐pH *C. ternatea* extract as a stable and multifunctional ingredient in lipid‐containing foods.

## Graphical abstract

pH significantly affects the extraction efficiency of TPC TAC and the extracted anthocyanin components. pH 3 and pH 7 yield the highest extraction efficiency and antioxidant activity. Extracts at pH 7 are suitable as blue color additives for neutral pH foods and as good antioxidants for fat‐containing gummies.

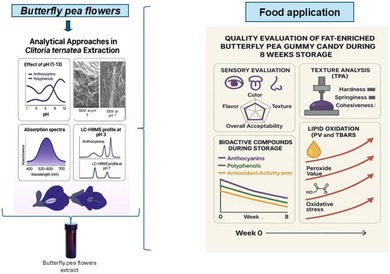

## 1 Introduction

*Clitoria ternatea* ( *C. ternatea* ), known as butterfly pea flower, belongs to the *Plantae* kingdom, *Tracheophyte* phylum, *Magnoliopsida* class, and *Fabaceae* family. It is recognized for its potential as a natural blue colorant for food, cosmetics, and pharmaceuticals (Jamil et al. 2018; Marpaung et al. 2017). Widely cultivated in tropical and humid climates, particularly in Southeast Asia (Mukherjee et al. 2008), the species demonstrates high adaptability and is commonly found throughout Vietnam. Its flowers, especially the petals, are rich in phenolic compounds and various bioactives, including alkaloids, tannins, glycosides, steroids, saponins, flavonoids, and phenols (Mohan 2013). Primary secondary metabolites include flavonol glycosides, myricetin, quercetin, phenolic acids, kaempferol, and anthocyanins (Jeyaraj et al. 2021; Vidana Gamage and Choo 2023).

Anthocyanins, notable for their vibrant pigmentation and safety in consumption, are valuable as natural food colorants. However, their application is limited by poor stability in aqueous environments, particularly under conditions involving variations in temperature, light, pH, oxygen, metal ions, and enzymatic activity (Houghton et al. 2021). Due to their structural fragility‐especially the unstable O1–C2 bond on the C‐ring‐anthocyanins are susceptible to degradation, leading to discoloration and loss of function during food processing and storage (Huang et al. 2021; Zang et al. 2021). The other studies have investigated anthocyanin stability across various pH and temperature conditions (Abdullah et al. 2010; Syahirah et al. 2018). Notably, the inclusion of stabilizing agents such as fructooligosaccharides has shown protective effects against photodegradation (Escher et al. 2020). Structural transformations of anthocyanins in response to pH shifts are closely linked to changes in extract coloration. While discoloration may not occur between pH 4–6, and no brown precipitate forms between pH 0.5–13 under varied temperatures, acidic extracts show higher storage stability compared to alkaline conditions. Red‐colored butterfly pea flower anthocyanin extract (CTAE) retained 70%–80% of its color over 60 days at 27°C–37°C and remained stable for over a year at 7 C (Abdullah et al. 2010).

The increasing demand for natural food additives has driven the exploration of plant‐derived alternatives to synthetic colorants, particularly those that provide both functional and aesthetic benefits. Among these, anthocyanins are a group of water‐soluble flavonoid pigments that have garnered significant attention due to their dual role as natural colorants and bioactive compounds with potent antioxidant properties. Despite their widespread occurrence in fruits and vegetables, most anthocyanins yield red to purple hues, and the development of stable blue coloration in food systems remains a persistent challenge, as blue pigments are rare in nature and tend to degrade under varying environmental conditions such as pH, light, and temperature (Giusti et al. 2023; Houghton et al. 2021). *C. ternatea* has emerged as a notable exception. This leguminous plant is rich in ternatins, a subclass of polyacylated anthocyanins capable of producing intense blue coloration. Unlike conventional anthocyanins, ternatins are more structurally stable under acidic conditions and exhibit enhanced thermal and photo‐induced degradation resistance, making them highly attractive for application in acidic food products such as beverages and confectionery (Chusak et al. 2018). Additionally, their amphiphilic nature—from semi‐polar glycosidic and acyl groups—facilitates partitioning into both aqueous and lipid environments, offering distinct advantages for use in fat‐containing functional foods (Enaru et al. 2021).

Lipid oxidation is a major challenge in food processing and preservation. It is a complex, self‐propagating reaction that produces reactive species such as hydroperoxides, aldehydes, and malondialdehyde (MDA), leading to the deterioration of oxidative stability, nutritional quality, and sensory attributes in lipid‐rich foods (Dobarganes and Velasco 2002; Halliwell and Gutteridge 1984). This process is catalyzed by heat, light, and transition metal ions, especially in matrices rich in unsaturated fatty acids. Numerous studies have demonstrated the efficacy of polyphenols and anthocyanins in mitigating lipid peroxidation through diverse mechanisms, including free radical scavenging, transition metal chelation, and inhibition of lipid‐oxidizing enzymes such as lipoxygenase (Repetto and Boveris 2012). In addition to their chemical activity, anthocyanins may interact with biopolymer matrices to form more stable complexes. Recent evidence suggests that anthocyanins can bind to polysaccharides such as pectin, gelatin, and agar via covalent and non‐covalent interactions, depending on the polymer structure and environmental conditions, thereby enhancing pigment retention and stability (Shi et al. 2024).

In this context, gummy candy was selected as a model system to evaluate the functionality of *C. ternatea* extract (CTE). This product type offers a polysaccharide‐rich matrix that supports anthocyanin–polymer interactions and mimics real‐world storage and processing conditions. The formulation allows assessment of anthocyanin stability and antioxidant capacity during storage and demonstrates the feasibility of using CTE as a dual‐purpose ingredient. It functions as a natural colorant and bioactive compound, supporting the development of clean labels and value‐added functional food products.

## 2 Materials and Methods

### 2.1 *C. ternatea* Flower

Fresh *Clitoria ternatea* flowers were collected between 06:00 and 08:00 AM from D'PALFARM Co. Ltd. (11°35′25″ N, 106°9′24″ E), located in Tay Ninh Province, Vietnam. The plant species was taxonomically identified at the Institute of Tropical Biology (Vietnam Academy of Science and Technology). After collection, the flowers were dried using a heat pump dryer at 41°C± 1°C until the moisture content reached 12%–13%. The dried samples were then packed in airtight polyethylene bags and stored under cool conditions (10°C–15°C, protected from light) prior to extraction. All samples were used within 1 month of drying. Before extraction, the dried flowers were ground into a coarse powder to ensure uniform extraction efficiency.

### 2.2 Chemicals & Equipments

#### 2.2.1 Chemical

Chemicals were purchased following: Gallic acid (Acros, Belgium), folin–ciocalteu (Sigma Aldrich, USA), sodium carbonate (Xilong, China), DPPH (2, 2‐diphenyl‐1‐picrylhydrazyl) (Acros, Belgium), Trolox (Sigma Aldrich, USA), sodium acetate (Xilong, China), potassium chloride (Xilong, China), concentrated hydrochloric acid (Xilong, China), HCl (Sigma Aldrich, USA), NaOH (Sigma Aldrich, USA).

#### 2.2.2 Equipments

Infrared Moisture Analyzer (ML‐50, AnD, Japan), convective drying (UM400, Memmert, Germany), pH machine (F20, Mettler Toledo, USA), pH meter equipped with a combined glass electrode suitable for nonaqueous systems (EtOH‐trode, Metrohm, Switzerland), thermostatic water bath (WNE‐29, Memmert, Germany), UV—Vis Spectrophotometer 25–1650‐01‐0406, vacuum pump (RV8, Edwards, Germany), Labconco FreeZone Freeze Dry System (USA), Q Exactive Hybrid Quadrupole‐Orbitrap Mass Spectrometer (Thermo Finnigan, San Jose, CA, USA).

### 2.3 Sample Preparation and Extraction

Dried butterfly pea flowers were processed following a previously validated extraction approach for recovery of anthocyanins and phenolic compounds, with minor modifications to enable evaluation across a wide pH range (Nguyen et al. 2025).

A portion (3 g) of dried butterfly pea flowers was homogenized and extracted using a hydroalcoholic solvent system (50% ethanol, v/v) at 50°C for 45 min. The extraction media were prepared by initially adjusting the pH of aqueous solutions (pH 1–13) using appropriate buffer systems (KCl–HCl for pH 1–2, acetate buffer for pH 3–6, phosphate buffer for pH 6–8, carbonate‐based buffer systems for pH 9–11, and NaOH‐based system for pH 12–13). The pH of each extraction medium was verified prior to extraction using a calibrated pH meter equipped with a combined glass electrode suitable for nonaqueous systems (EtOH‐trode, Metrohm, Switzerland), ensuring reliable pH measurement in ethanol‐containing media. After extraction, the mixtures were filtered under vacuum and concentrated using a rotary evaporator (IKA RV 3 Series, Germany) at 45°C and 60 rpm for 60 min to obtain concentrated extracts (CTE) with a final moisture content of approximately 35%–37%.

### 2.4 Chemical Assays

#### 2.4.1 TPC, Tac, DPPH

TPC value was determined using the Folin‐Ciocalteau colorimetric assay, following the general principles described by (Agbor et al. 2014) with slight modifications. Briefly, 0.5 mL of appropriately diluted extract was mixed with 2.5 mL of 10‐fold diluted Folin–Ciocalteu reagent and allowed to react for 5 min. Subsequently, 2.0 mL of sodium carbonate solution (7.5%, w/v) was added. The mixture was incubated at room temperature in the dark for 30 min, and absorbance was measured at 765 nm using a UV–Vis spectrophotometer. Gallic acid was used for the calibration curve, and results were expressed as mg gallic acid equivalents per g dry matter (mg GAE/g DM).

TAC value is determined by the differential pH method (Fuleki and Francis 1968). Extracts were diluted separately in potassium chloride buffer (0.025 M, pH 1.0) and sodium acetate buffer (0.4 M, pH 4.5). Absorbance was recorded at 520 and 700 nm using a UV–Vis spectrophotometer. TAC values were calculated as cyanidin‐3‐glucoside equivalents using a molecular weight of 449.2 g/mol and a molar absorptivity of 26,900 L·mol^−1^·cm^−1^ and expressed as mg cyanidin‐3‐glucoside equivalents per g dry matter.

Antioxidant activity was evaluated using the 2, 2‐diphenyl‐1‐picrylhydrazyl (DPPH) radical scavenging assay, following the procedure referenced (Brand‐Williams et al. 1995). A DPPH solution (0.1 mM) was prepared in methanol. An aliquot of 0.1 mL extract was mixed with 3.9 mL DPPH solution and incubated for 30 min in the dark at room temperature. Absorbance was measured at 517 nm. Trolox was used as a standard, and results were expressed as μmol Trolox equivalents per g dry matter (μmol TE/g DM).

All measurements were conducted in triplicate.

### 2.5 Structural and Spectral Analysis

#### 2.5.1 UV–Vis's Spectroscopy

UV–Vis spectral analysis of *C. ternatea* extracts adjusted to pH values ranging from 1 to 13 was performed using a spectrophotometer (Model 25–1650‐01‐0406). Absorbance was measured across the 250–700 nm wavelength range in absorbance mode, with a spectral resolution interval of 0.2 nm using 1 cm quartz cuvettes, with distilled water as blank. Samples were appropriately diluted to ensure absorbance values within the linear detection range. Spectral interpretation of anthocyanin absorption characteristics and pH‐dependent shifts was based on previously established anthocyanin spectroscopic behavior (Giusti and Wrolstad 2001).

#### 2.5.2 Scanning Electron Microscopy (SEM)

Residual solids from extractions at pH 3 and pH 7 were dried to ~5% moisture, mounted on aluminum stubs using double‐sided conductive tape, and sputter‐coated with a thin gold layer. Surface morphology was observed using a conventional scanning electron microscope operated in high‐vacuum mode with a secondary electron detector (SED) at an accelerating voltage of 10 kV and a working distance of approximately 10.8 mm. Micrographs were recorded at magnifications ranging from × 100 to × 10,000 to evaluate cell‐wall disruption and structural changes.

#### 2.5.3 LC–HRMS Profiling

Anthocyanin profiling was performed using a Dionex Ultimate 3000 high‐performance liquid chromatography (HPLC) system (Thermo Finnigan, San Jose, CA, USA) coupled with a Q Exactive Hybrid Quadrupole‐Orbitrap Mass Spectrometer (Thermo Finnigan, San Jose, CA, USA). Chromatographic separation was carried out on a Luna C18 column (150 mm × 2.00 mm, 5 μm particle size; Phenomenex, USA) maintained at 30°C. The mobile phase consisted of solvent A (acetonitrile) and solvent B (0.05% v/v formic acid in water), with a gradient elution program as follows: 0–10 min, 98% B; 10–18 min, 80% B; 18–25 min, 40% B; and 25–30 min, re‐equilibration to 98% B. The flow rate was set to 0.3 mL/min. All data acquisition and processing were conducted using Thermo Scientific Xcalibur software.

**Q‐Orbitrap Mass Spectrometry Conditions:** The Q Exactive mass spectrometer was operated in positive electrospray ionization (ESI) mode with a full MS scan range of *m/z* 100–1500 and a resolution of 70,000 (FWHM). Nitrogen was used as the sheath and auxiliary gas. The HESI parameters were as follows: sheath gas flow rate, 30 arbitrary units (arb); auxiliary gas, 10 arb; sweep gas, 0 arb; S‐lens RF level, 50 V; capillary temperature, 32 C; and auxiliary gas heater temperature, 30°C. MS/MS fragmentation was performed using higher‐energy collisional dissociation (HCD) with a normalized collision energy (NCE) of 20. Nitrogen was also used as the collision and spray stabilization gas within the C‐trap. External mass calibration was performed prior to analysis, and mass accuracy was maintained within 5 ppm. Identification of anthocyanin compounds was based on their fragmentation patterns in comparison with previously reported reference spectra. Semi‐quantitative analysis was performed by calculating the relative abundance of individual anthocyanins as the percentage of their respective peak areas in the total ion chromatogram (TIC).

### 2.6 Fat‐Enriched Gummy Candy Application

#### 2.6.1 Gummy Candy Preparation

Gelatin was soaked in water at room temperature for 30 min and then dissolved by heating at 80°C. In parallel, sugar and malt syrup were dissolved in water and heated to 80°C until homogeneous, after which the gelatin solution was incorporated. The mixture was cooled to approximately 60°C, and Anchor butter (82.9% fat) was added at concentrations of 0, 3, 6, or 9% (w/w, relative to the total mixture), together with *C. ternatea* extract (CTE) at 0, 0.5, 1.0, 1.5 or 2.0% (w/w). CTE served both as a natural blue colorant and as a source of antioxidant activity. After gentle mixing, citric acid was added to adjust flavor and enhance stability. The final mixture was poured into pre‐cooled silicone molds at 4°C, allowed to set for 3–4 h, demolded, and further conditioned at 4°C for 20 h. The gummies were then packaged in vacuum‐sealed PE–PA (polyethylene–polyamide) pouches.

The finished products were analyzed for total anthocyanin content (TAC, mg/g candy), total polyphenol content (TPC, mg/g candy), and DPPH antioxidant activity (μmol Trolox equivalents/g candy). Lipid oxidation was monitored using peroxide value (PV) and thiobarbituric acid reactive substances (TBARS) as indicators. All parameters were measured during an 8–week storage period at 30°C. Each formulation was prepared and analyzed in triplicate to ensure reproducibility.

#### 2.6.2 Sensory Evaluation

Sensory evaluation was conducted using a 5‐point hedonic scale (1 = dislike extremely, 5 = like extremely) by 20 semi‐trained assessors under standardized lighting and serving conditions. Panelists were familiar with the evaluation procedure prior to assessment. The evaluated attributes included color, flavor, taste, texture, and overall acceptability.

### 2.7 Texture Profile Analysis (TPA)

The structural properties of the gummy samples were evaluated using a texture analyzer (TA‐XT Plus, Stable Micro Systems, UK) connected to a Windows‐based computer running Exponent Connect Lite version 7.0. TPA was performed under the following conditions: a compression force of 25 kg, a test speed of 1 mm/s, and a strain level set to 50% of the sample height (Bourne 1978).

### 2.8 Lipid Oxidation Indices

Peroxide value (PV) was determined following the iodometric titration method involving acetic acid–isooctane solvent with potassium iodide and titration with sodium thiosulfate using starch as the endpoint indicator (ISO‐3960 2017).

### 2.9 Thiobarbituric Acid Reactive Substances (TBARS)

TBARS is measured based on the determination of malondialdehyde (MDA) content using the TBARS index. The principle of the TBARS assay relies on the reaction between MDA and thiobarbituric acid (TBA), forming a pink‐colored complex that exhibits strong absorbance at approximately 532 nm. The absorbance intensity is directly proportional to the concentration of MDA, thereby reflecting the extent of lipid peroxidation in the sample. This method was conducted according to (Prasetyo et al. 2008).

### 2.10 Presenting Research Results

All experiments were performed at least three times to calculate the average results. The results are presented as mean ± standard deviation. The experimental results were processed by One‐way Analysis of Variance (ANOVA) with Statgraphics Centurion 19 (version X64) software. Data was expressed as mean ± standard deviation (*n* = 3). The significant differences between the experimental results were compared by Multiple range tests (*p* < 0.05). Tukeys HSD post hoc multiple comparison test was applied to determine pairwise differences among means.

## 3 Results and Discussion

### 3.1 The Effect of pH on TAC, TPC, and DPPH Antioxidant Activity

**FIGURE 1:**
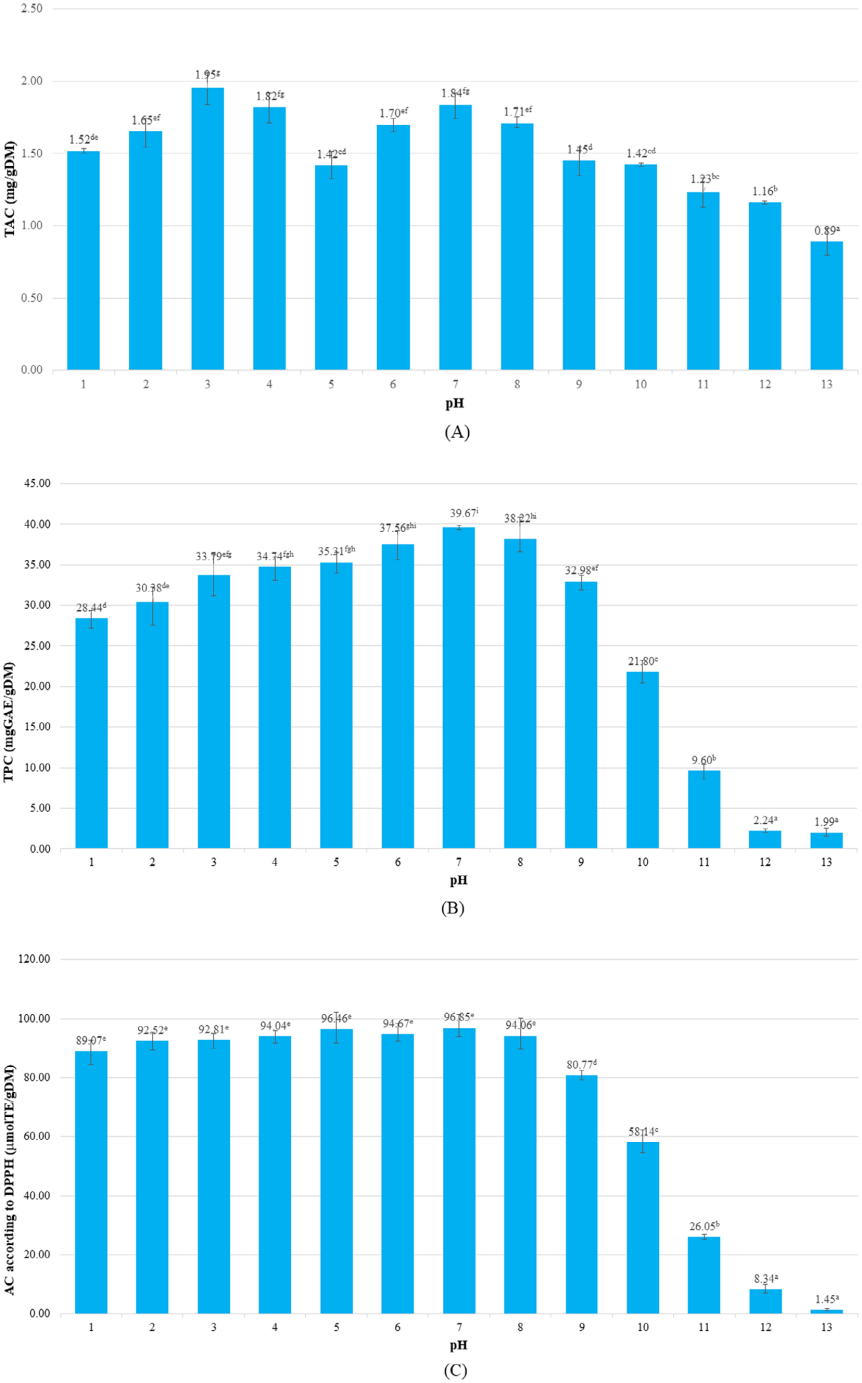
(A, B, C): The effect of pH on TAC, TPC, and DPPH antioxidant activity. Different letters above the columns indicate statistically significant differences (*p* < 0.05). Values are presented as mean ± standard deviation (*n* = 3). DM, dry matter.

Fresh *C. ternatea* flowers obtained from D PALFARM Co. Ltd. (Tay Ninh Province, Vietnam; 11°35′25″ N, 106°9′24″ E) were processed as described in Section 2.3 and extracted over a pH range of 1–13. The extracts were subsequently analyzed for total anthocyanin content (TAC), total polyphenol content (TPC), and DPPH antioxidant activity. The results are shown in Figure 1.

Figure 1A showed that the highest TAC was observed at pH 3 and pH 7, indicating that anthocyanins were more stable in high acidic (pH 3) and neutral (pH 7) conditions. At pH 3, anthocyanins preserve their molecular structure, color, and contribute to higher TAC levels. At pH 7, although less stable than at pH 3, anthocyanins still maintain significant antioxidant activity, with TAC remaining relatively high. At intermediate pH values (around pH 5), TAC decreased noticeably. Anthocyanin instability at pH 5 can be explained by its position within the hydration–hemiketal equilibrium region. At this pH, the flavylium cation partially converts to colorless hemiketal and chalcone forms, reducing chromophore integrity and apparent TAC. In contrast, at alkaline pH (9_13), the stability of anthocyanins declines significantly, leading to a considerable reduction in TAC due to molecular breakdown, color loss, and diminished antioxidant capacity. These findings are consistent with previous studies by (Giusti and Wrolstad 2001) and (Lee et al. 2002), which highlight the enhanced stability of anthocyanins in acidic to neutral pH environments. Figure 1B showed that the TPC in butterfly pea flower extract was evaluated across a pH range from 1 to 13. The results indicated that TPC was significantly higher in the slightly acidic to neutral pH range (4_7), with the maximum content being observed at pH 7 (39.667 mg GAE/g CK). At lower pH values (1–3), TPC was notably lower, ranging from 8.439 mg GAE/g CK to 30.375 mg GAE/g CK, suggesting that acidic environments negatively affected polyphenol stability and extraction. In contrast, a significant reduction in TPC was observed at higher pH values (8–13), with the lowest value recorded at pH 13 (1.990 mg GAE/g CK). This trend is consistent with previous findings showing that the stability of phenolic compounds is highly dependent on pH. Several common phenolics, such as chlorogenic acid, caffeic acid, and gallic acid, undergo structural degradation under highly acidic or alkaline conditions, with the most pronounced instability observed at extreme pH levels. In contrast, these compounds tend to remain more stable within a moderate pH range, particularly between pH 4 and 7, which facilitates both their extraction and preservation (Friedman and Jürgens 2000). Figure 1C showed that the highest DPPH antioxidant activity was observed in extracts obtained at pH values between 1 and 7. However, as the pH increased beyond 7, a gradual decrease in antioxidant activity was observed, with the most significant loss occurring between pH 11 and 13. This is likely due to the degradation of sensitive polyphenolic structures. Alkaline conditions promote the oxidation and decomposition of polyphenols, leading to diminished functional properties, including reduced antioxidant activity and pigment loss. Therefore, the optimal pH for extracting and retaining polyphenols from *C. ternatea* appears to fall within the mildly acidic to near‐neutral range (pH 4–7), consistent with the pattern described (Friedman and Jürgens 2000).

The results from Figure 1 indicate that anthocyanins and polyphenols in *C. ternatea* exhibited the highest stability at pH 3 and pH 7, with the strongest antioxidant activity observed under slightly acidic to neutral conditions. In contrast, alkaline environments (pH 9–13) significantly reduce both the stability and antioxidant capacity of these compounds. These findings are consistent with previous studies, suggesting that the pH range of 4–7 is optimal for the extraction of polyphenolic and anthocyanin compounds from butterfly pea flower (Fu et al. 2021).

The quantitative results demonstrate that extraction efficiency and compositional profiles vary with pH in a compound‐specific manner. Maximum TAC at pH 3 reflects stabilization of the flavylium chromophore, whereas peak TPC and DPPH antioxidant activity near pH 7 indicate greater recovery of non‐anthocyanin phenolics and transformed anthocyanin derivatives. These numerical trends show that extraction behavior arises from combined effects of pigment stability, solubility, and pH‐driven structural changes across phenolic classes. For detailed molecular characterization, pH selection was guided by chemical representativeness rather than extraction yield alone. pH 3 and pH 7 correspond to two comparatively stable anthocyanin domains the flavylium cation form and the partially stabilized quinoidal/neutral form—where chromophore integrity and molecular identity remain sufficiently preserved for reliable profiling. In contrast, intermediate and alkaline pH conditions favor rapid conversion to chalcone structures, hydration products, and degradation intermediates. Such dynamic transformations complicate LC‐HRMS interpretation and do not reflect compositional states relevant to food applications. Based on these results, further analyses were conducted, including SEM imaging to observe cellular structure, UV–VIS spectroscopy to determine absorption wavelengths, and LC‐HRMS chromatography to identify anthocyanin compounds at pH 3 and pH 7. These advanced characterizations provide a foundation for applying *C. ternatea* in food‐related applications.

### 3.2 SEM

**FIGURE 2:**
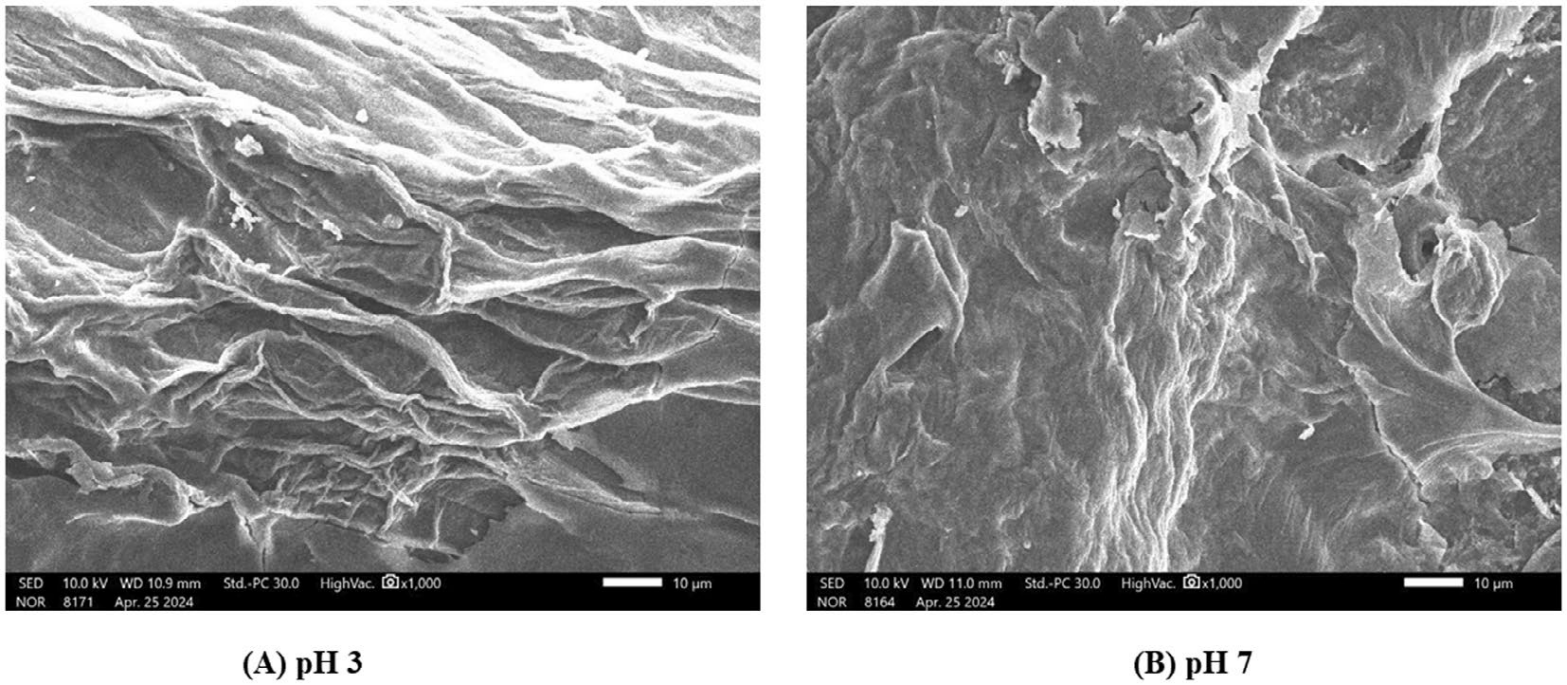
(A, B): SEM images of *C. ternatea* flower samples obtained at pH 3 and pH 7.

SEM imaging was performed on dried plant material obtained from *CTE* at pH 3 and pH 7, as prepared according to Section 2.5.2. The microstructural differences observed are shown in Figure 2.

Figure 2 revealed a pronounced effect of pH on the cell wall structure of *C. ternatea* petals. At pH 3 (Figure 2A), the cell wall was more extensively disrupted compared to pH 7 (Figure 2B). Specifically, cells exhibited signs of delamination, surface folding, and loss of structural integrity, whereas at pH 7, the cell surfaces remained largely intact. This difference is primarily attributed to the acidic environment, which promotes protonation of carboxyl groups on pectin, particularly within the homogalacturonan (HG) regions. This protonation leads to the dissociation of calcium‐mediated cross‐links (Ca^2+^–pectin), thereby destabilizing the polysaccharide matrix that maintains cell wall integrity (Phyo et al. 2019). In addition, low pH conditions enhance cellulose hydration and disrupt hydrogen bonding with hemicellulose, resulting in a more “fluid” and collapsible cell wall structure (Cosgrove 2000). This structural instability is consistent with the tissue disintegration observed in the SEM images. These findings underscore that extraction pH influences not only the efficiency of bioactive compound recovery but also the degree of microstructural damage to plant tissues.

### 3.3 UV–Vis Absorption Profiles

**FIGURE 3:**
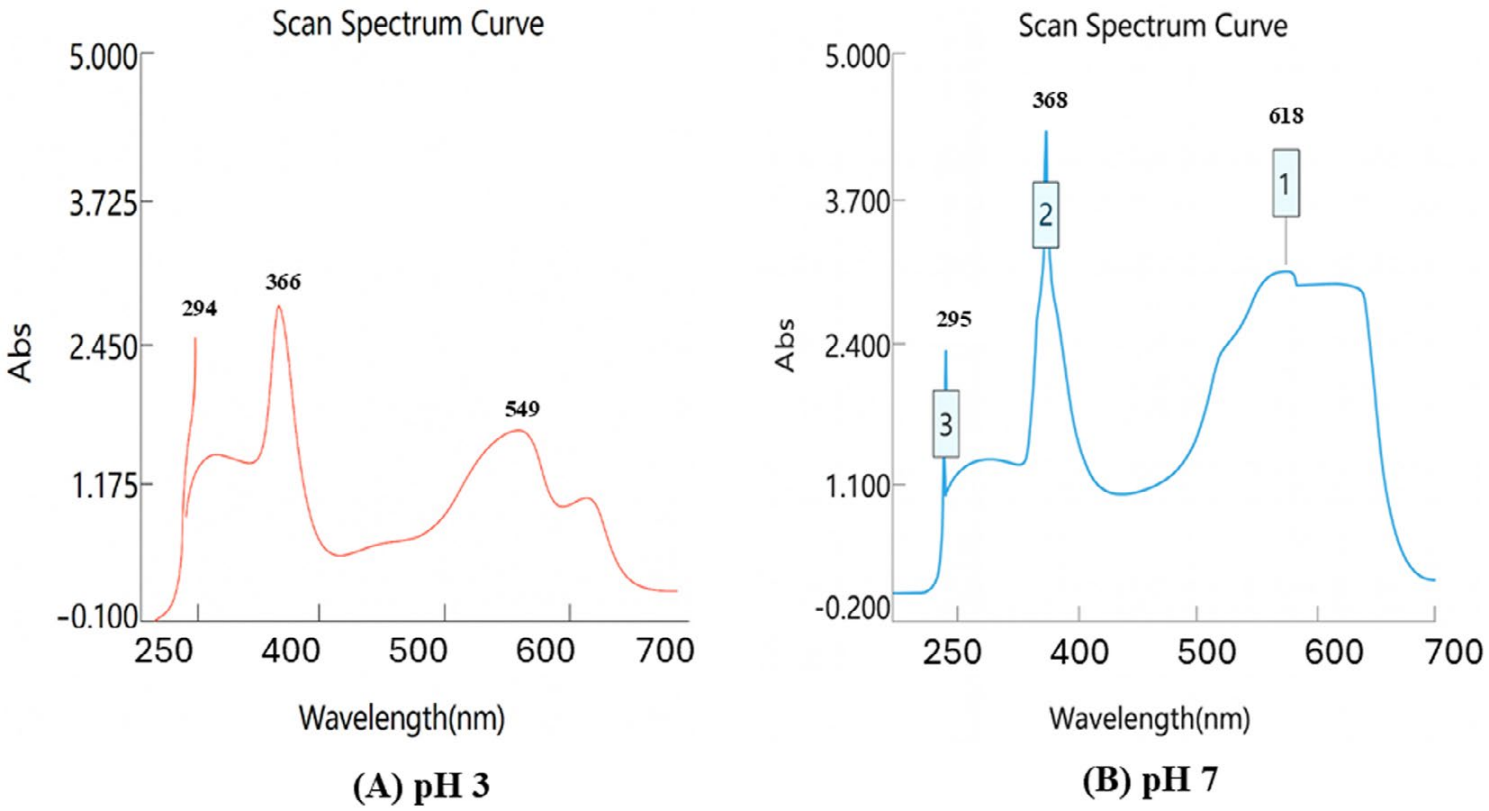
(A, B): Absorption spectra of CTE at pH 3 and pH 7.

The absorption spectra of CTE at pH 3 and pH 7 were shown in Figure 3 below:

At pH 3 (Figure 3A), the CTAE exhibited a purplish‐red color. Three absorbance peaks were observed at 294 nm, 366 nm, and 549 nm, with the maximum absorbance occurring at 549 nm. At pH 7, the CTAE appeared bright blue, showing peaks at 295.4 nm, 368 nm, and 618 nm, with the maximum absorbance recorded at 581 nm (Absorbance = 2.911). The UV–Vis spectra of the extracts showed characteristic absorption in the visible region (~520–550 nm), corresponding to anthocyanin chromophores, which is consistent with the observed color intensity and TAC values.

These UV–Vis results are consistent with previous studies regarding the pH‐dependent stability of anthocyanins. At pH 3, anthocyanins are stable in the flavylium cation form, exhibiting strong absorption in the 520–550 nm range. At pH 7, a bathochromic shift occurs, with the absorbance peak shifting to the 570–620 nm range, indicating a structural transformation into the quinonoidal base form (Marpaung 2020).

### 3.4 Anthocyanin Composition by LC–HRMS

**FIGURE 4:**
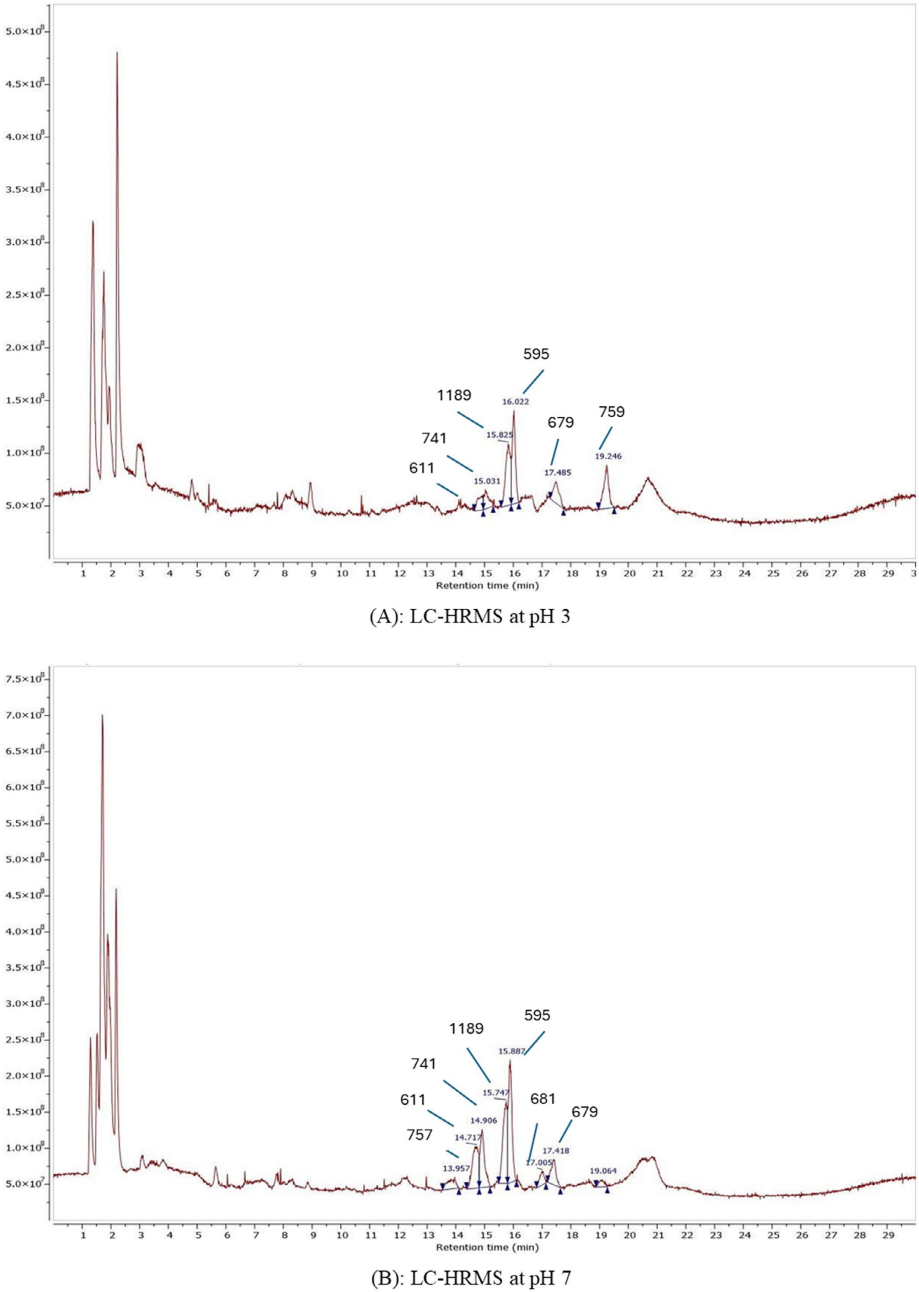
LC‐HRMS analysis of CTE at pH 3 & pH 7.

The LC‐HRMS analysis was conducted to compare the effect of different treatments on the composition of *C. ternatea* . The identity of phytochemicals was verified based on their MS/MS fragmentation and comparison with literature data. The concentrations of *C. ternatea* flower extracts were determined based on the percentage of peak area. The results are presented in Table 1, Figure 4 of CTE by LC‐HRMS at pH 3 & pH 7. Seven compounds belonging to the anthocyanin group as well as the aglycone of quercetin and kaempferol were identified in different treatments.

**TABLE 1:** Composition of anthocyanin compounds tentatively identified by LC‐HRMS at pH 3 and pH 7.

No	Molecular ion [M + H]^+^	pH 3		pH 7		MS^2^	Tentative identification	References
		Retention time (minutes)	Concentration (%)	Retention time (minutes)	Concentration (%)			
**1**	757			13.975	28.31	465 [M + H‐146−146]^+^ 303 [M + H‐146−146−162]^+^	Quercetin 3‐[2G]‐rhamnosylrutinoside	(Kazuma et al. 2003b; Lin and Harnly 2007; Silalahi 2020)
**2**	611	14.235	27.53	14.717	22.34	456 [M + H‐146]^+^ 303 [M + H‐146−162]^+^	Delphinidin‐3‐(cis‐p‐coumaroyl‐glucoside)	(Bationo et al. 2023; Thuy et al. 2021)
**3**	741	15.031	7.63	14.906	15.56	449 [M + H‐146−146]^+^ 287 [M + H‐146−146−162]^+^	Kaempferol 3‐(6″‐p‐coumaroyl)‐rutinoside	(Kazuma et al. 2003a; Tatsuzawa et al. 2014)
**4**	1189	15.825	6.80	15.747	4.86	449, 287, 271, 182	Unknown (could correspond to kaempferol derivative)	
**5**	595	16.022	11.04	15.887	15.81	449 [M + H‐146]^+^ 287 [M + H‐146−162]^+^	Cyanidin‐3‐(p‐coumaroyl)glucose	(Thuy et al. 2021)
**6**	681			17.005	3.60	535 [M + H‐146]^+^ 287 [M + H‐146−86−162]^+^	Kaempferol‐rhamnosyl‐malonyl‐glucoside	(Jeyaraj et al. 2022; Kazuma et al. 2003a; Szewczyk et al. 2016)
**7**	679	17.485	27.92	17.481	2.30	679, 404, 287, 212, 147	Unknown (could correspond to aglycoside cyanidin)	
**8**	759	19.246	18.72	19.064	7.22	741, 359, 331, 212	Unknown (no previous data have been reported)	

Compound 1 presented ion [M + H] + m/z at 757, producing the fragment with m/z at 465, showing a loss of 146 Da, and 308 Da which was given from aglycone m/z 303. Based on the reference of previous studies, compound 1 was assigned to Quercetin 3‐[2G]‐rhamnosylrutinoside (Kazuma et al. 2003a; Lin and Harnly 2007; Silalahi 2020). Compound 2 presented [M + H] + at m/z 611 and yielded similar ion fragmentation to compound 1 of m/z at 303. However, the mass spectrum showed the presence of a fragment ion m/z 147, corresponding to coumaroyl. Thus, compared with the data and the findings of (Thuy et al. 2021), compound 2 was suggested as Delphinidin‐3‐(p‐coumaroyl‐glucoside) (Bationo et al. 2023; Thuy et al. 2021). Compound 3 was designated Kaempferol 3‐(6‐p‐coumaroyl)‐rutinoside due to showing protonated ion m/z at 741 and producing some fragments such as m/z 449, 287, evidencing the loss of two of neutral ions (146 Da) and glucoside group (162 Da). In addition, fragment ion 147 Da suggested this compound contained coumaroyl (Kazuma et al. 2003a; Tatsuzawa et al. 2014). Compound 4 presented [M + H] + with m/z at 1189 and produced several characteristic fragment ions at m/z 449, 287, 271, and 182. The fragment at m/z 287 could correspond to the aglycone cyanidin or kaempferol, which is consistent with a neutral loss of 162 Da from the fragment at m/z 449. However, the available data were insufficient to unambiguously determine the identity of the compound with a molecular weight of 1189. Compound 5 showed protonated ion with [M + H] + m/z 595, which was indentified Cyanidin‐3‐(p‐coumaroyl) glucose by (Thuy et al. 2021). Moreover, its fragments m/z 449, 287, 147 revealed this compound involving coumaroyl (147 Da), glucoside (loss of neutral ion [M + H146‐162]+), and aglycone cyanidin (287 Da) (Thuy et al. 2021). Compound 6 was designated as Kaempferol‐rhamnosyl‐malonyl‐glucoside due to its protonated ion [M + H] + m/z at 681. Furthermore, its fragmentation showed ion m/z at 535, 287, evidencing malonyl, hexose unit, and aglycone kaempferol (Jeyaraj et al. 2021; Kazuma et al. 2003a; Szewczyk et al. 2016). Compound 7 showed [M + H] + ion m/z at 679 exhibited a protonated molecular ion [M + H]^+^ at m/z 679 and generated fragment ions at m/z 404, 287, 212, and 147. The fragment at m/z 287 may correspond to the aglycone cyanidin or kaempferol. Compound 8 presented [M + H] + at m/z 759 and produced fragment ions at m/z 741, 359, 331, and 212; however, no previous data have been reported to support the identification of this compound. To date, no relevant literature evidence has been found to substantiate the structural assignment of these two compounds. Consequently, their identities remain inconclusive, and further analytical studies are required to precisely elucidate their chemical structures.

The acylated anthocyanins detected in this study belong to the structural class commonly described as ternatin‐type pigments in *C. ternatea* . These molecules contain multiple aromatic acyl substituents (e. g., p‐coumaroyl groups) attached to glycosyl chains, which promote intramolecular co‐pigmentation through π–π stacking interactions. This structural feature provides steric shielding of the flavylium chromophore and contributes to enhanced stability compared with non‐acylated anthocyanins. The detection of these acylated derivatives at pH 3 and pH 7 is consistent with their known stability in acidic to near‐neutral environments, where chromophore integrity can be preserved. Under strongly alkaline conditions, however, even polyacylated anthocyanins are susceptible to hydration, chalcone formation, and subsequent degradation, which limits their detectability and practical relevance in food systems.

There was a variety of phytochemicals tentatively identified in *C. ternatea;* therein, flavonols and anthocyanins were two of the subclasses belonging to the flavonoid group of polyphenol compounds, which were noted not only in coloring but also for their therapeutic potential. Anthocyanins such as ternatin A, B, C, D were commonly found in water or ethanol extraction but were not present in this study. The reason could be their origin, similar to the study of (Thuy et al. 2021) only found that delphinidin‐3‐(cis‐p‐coumaroyl‐glucoside), cyanidin‐3‐(p‐coumaroyl) glucose, and delphinidin glucoside in *C. ternatea* , originating from Vietnam (Jeyaraj et al. 2022; Thuy et al. 2021; Vidana Gamage and Choo 2023). In this study, the cyanidin‐3‐(p‐coumaroyl) glucose compound is present in most treatments and was the most abundant compound in butterfly flower extract.

The comparison between LC–MS and UV–Vis data at pH 3 and pH 7 indicates that anthocyanins were more abundant at pH 3. It could be pH causing unstable flavonols and anthocyanins in CTE, although previous studies showed pH was a significant factor influencing both color and some bioactivities such as antioxidants without significantly affecting the concentration (Kungsuwan et al. 2017). In this study, quercetin and kaempferol derivatives also appeared in the extract, tending to increase health benefits such as antioxidant, anti‐inflammatory activity, and antihypertensive for this material. Moreover, the UV–Vis spectrum at pH 3 exhibited a strong absorption maximum of around 549 nm, corresponding to the flavylium cation form responsible for the characteristic red‐purple color of anthocyanins. In contrast, at pH 7, a blue‐shifted absorption peak at 618.6 nm suggests structural transformation into quinoidal base or other hydrated forms, which are typically less visually stable (Vidana Gamage and Choo 2023).

The variation in anthocyanin compounds in CTE at pH 3 and pH 7 can be attributed to chemical stability and reactivity shifts under different pH conditions. At pH 3, anthocyanins predominantly exist in the flavylium ion form, which is more stable but less chemically reactive with sugar and acyl groups. This stability limits the formation of glycosylated and acylated flavonoids such as Quercetin 3‐[2G]‐rhamnosylrutinoside and Kaempferol‐rhamnosyl‐malonyl‐glucoside. In contrast, at pH 7, anthocyanins transition into their quinoidal base forms, which exhibit greater reactivity, allowing a relatively greater contribution and detectability of co‐extracted flavonol glycosides and acylated derivatives. The neutral to slightly alkaline environment thus promotes the presence of highly substituted flavonoids absent in acidic conditions, accounting for the observed differences in compound profiles between the 2 pH levels (Giusti and Wrolstad 2001; Kazuma et al. 2003a; Lee et al. 2002). The differences in compound profiles observed between pH 3 and pH 7 are therefore attributed primarily to pH‐dependent structural equilibria and stability rather than new chemical formation. At pH 3, anthocyanins predominantly exist as the flavylium cation, which preserves chromophore integrity and favors detection of intact pigments. At pH 7, partial conversion to quinoidal base forms and increased susceptibility to hydration may reduce the apparent abundance of some anthocyanins, while allowing relatively greater proportional contribution of co‐extracted flavonol glycosides such as quercetin and kaempferol derivatives. These compositional differences reflect shifts in molecular stability and spectral behavior rather than pH‐induced synthesis or transformation into new flavonoid structures. This interpretation also explains the reduced TAC observed at pH 5 in the present study, where pigment structural equilibrium shifts away from chromophore‐dominant forms, resulting in lower detectable anthocyanin levels compared with pH 3 and pH 7.

In agreement with LC‐HRMS results, the extract at pH 7 exhibited a broader polyphenolic profile, including flavonol glycosides and acylated derivatives, which are associated with enhanced antioxidant potential and functional properties. The combined interpretation of TAC, UV–Vis spectra, and LC‐HRMS profiles therefore highlights that pH not only influences anthocyanin stability but also modulates the overall composition and functional potential of the extract.

### 3.5 Application of Neutral‐pH CTE in Gummy Candy

#### 3.5.1 Formulations of *C. ternatea* Gummy Candy

The acylation of flavonoid glycosides, as tentatively identified in the LC‐HRMS results, helps explain the retention of the natural color integrity of *CTE*. Building on these findings, the next phase of this study applied CTE, prepared under its natural extraction pH (~6.8–7.0), into a food matrix composed primarily of glucose syrup, gelatin, and agar. These components promote the formation of flavonoid–polymer complexes, resulting in a structural network that can encapsulate anthocyanin molecules or limit their exposure to oxygen and metal ions, thereby enhancing color stability (Duan et al. 2022).

CTE was incorporated into enriched gummy candy as a natural colorant and antioxidant source, enriching the product with bioactive compounds. This study optimized ingredient ratios for gummy production and assessed product quality based on moisture content, pH, total sugar, reducing sugar, TAC, TPC, and DPPH antioxidant activity during two months of storage. The CTE composition is presented in Table 2.

**TABLE 2:** Chemical composition of CTE.

Characteristic	Unit	CTE	Method
Moisture	%	36.06 ± 0.98	QTTN/KT3 136:2016
Protein content	g/g DM	0.339 ± 0.012	Kjeldahl method
Carbohydrate content	g/g DM	0.651 ± 0.003	QTTN/KT3 317:2022
Ash content	%	0.345 ± 0.013	AOAC 942.05, 923.03
TPC	mgGAE/g DM	135.81 ± 0.081	ISO 14502‐1:2005
TAC	mg/g DM	6.81 ± 0.005	AOAC 2005.02

The selected concentration ranges of CTE (0%–1.5%, w/w) and fat (0%–9%, w/w) were established through preliminary formulation screening trials conducted to define a technically feasible experimental window for gummy candy preparation. In the initial trials, higher CTE levels resulted in excessively dark color and slight astringency, whereas higher fat levels caused greasy mouthfeel, phase separation, and reduced gel uniformity. Therefore, the selected ranges were considered suitable for evaluating the combined effects of CTE and fat on product quality and oxidative stability. The corresponding gummy formulations used in this study are summarized in Table 3, while detailed results of the preliminary screening are provided in Table S1, which shows that formulations exceeding 1.5% CTE or 9% fat resulted in unacceptable sensory and structural properties.

**TABLE 3:** Formulations of CTE gummy candy.

Ingredients	%
Sugar	32.5–33.0
Glucose syrup	10.4–11.0
Gelatin	11.0
Water	35.0–40.0
CTE	0–2.0
Anchor butter	0–9.0

#### 3.5.2 Sensory Evaluation

Based on the technically feasible concentration ranges identified in the preliminary screening, a formal sensory evaluation was conducted to determine the optimal levels of CTE and butter in the gummy formulation. Gummy samples were prepared with varying concentrations of CTE (0%, 0.5%, 1.0%, 1.5% and 2.0%, w/w) and butter (0%, 3, 6%, and 9%, w/w).

**FIGURE 5:**
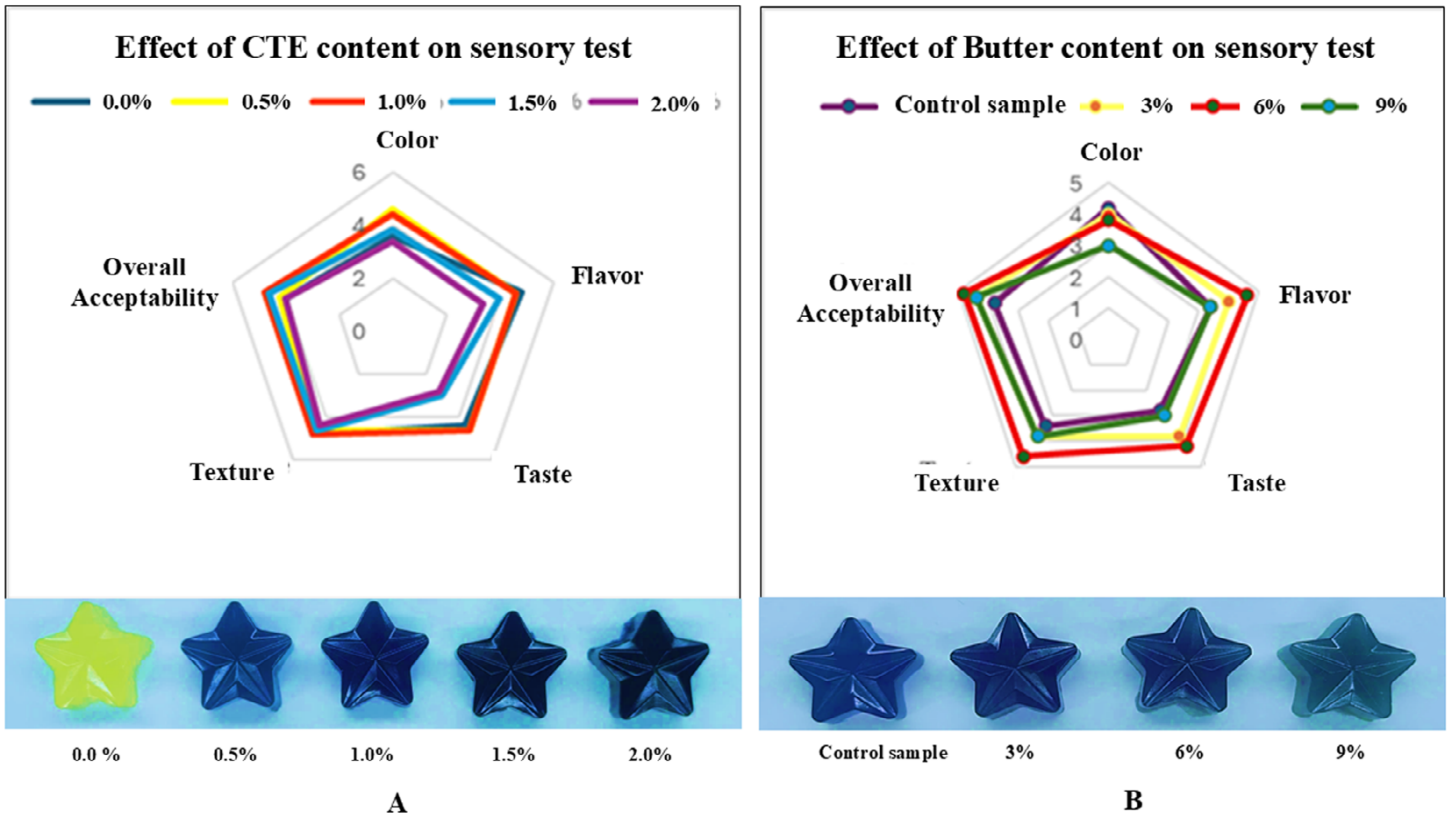
(A, B): Sensory evaluation of gummy candies enriched with CTE and butter at different levels.

Sensory evaluation results indicated that the concentration of CTE and fat content significantly affected the sensory quality of gummy candy (Figure 5A,B).

Sensory evaluation results indicated that the concentration of CTE and fat content significantly affected the sensory quality of gummy candy (Figure 5A,B). In the CTE‐supplemented groups, samples containing 0.5% and 1.0% CTE received significantly higher scores in terms of color, flavor, taste, and overall acceptability compared to those with 1.5% and 2.0% CTE (*p* < 0.05), which were characterized by darker coloration and a slightly astringent taste. In contrast, texture scores remained consistent across all CTE levels, suggesting that CTE content did not significantly affect the structural integrity of the gummies. Overall, 1.0% CTE was identified as optimal, offering a desirable balance of natural color, palatable flavor, and acceptable texture. Regarding fat content, the sample containing 6% Anchor butter achieved the highest sensory scores for taste (4.20 ± 0.55), texture (4.60 ± 0.55), and overall acceptability (4.80 ± 0.50), indicating improved chewiness and a more harmonious buttery flavor. While the 3% fat sample provided a pleasant aroma, its taste and texture were not considered optimal. In contrast, the 9% fat sample received lower scores due to a greasy odor and increased firmness. Notably, the 6% fat formulation did not negatively affect anthocyanin color stability and was perceived to maintain the best overall sensory quality. Based on these findings, the optimal formulation was determined to contain 1.0% CTE and 6.0% butter and was selected for further evaluation of physicochemical parameters, including TPA, TAC, TPC, DPPH antioxidant activity, PV, and TBARS during a 60‐day storage period.

#### 3.5.3 Texture Analysis by TPA After 8‐Week Storage Period

Since sensory evaluation indicated that formulations containing more than 1.0% CTE were less acceptable, texture profile analysis was focused on the lower and practically relevant concentration range (0%–1.0%). Intermediate concentrations (0.2% and 0.6%) were included to provide a clearer assessment of the dose‐dependent effects of CTE on the mechanical properties of the gummy matrix.

Textural properties, including hardness, springiness, adhesiveness, and chewiness, were evaluated at week 0 and week 8 using a TPA instrument to assess the influence of CTE incorporation on the mechanical structure of the gummy system during storage. Formulations containing 0.0%, 0.2%, 0.6%, and 1.0% CTE (w/w) were prepared with a fixed butter content of 6%, as established by sensory optimization.

**FIGURE 6:**
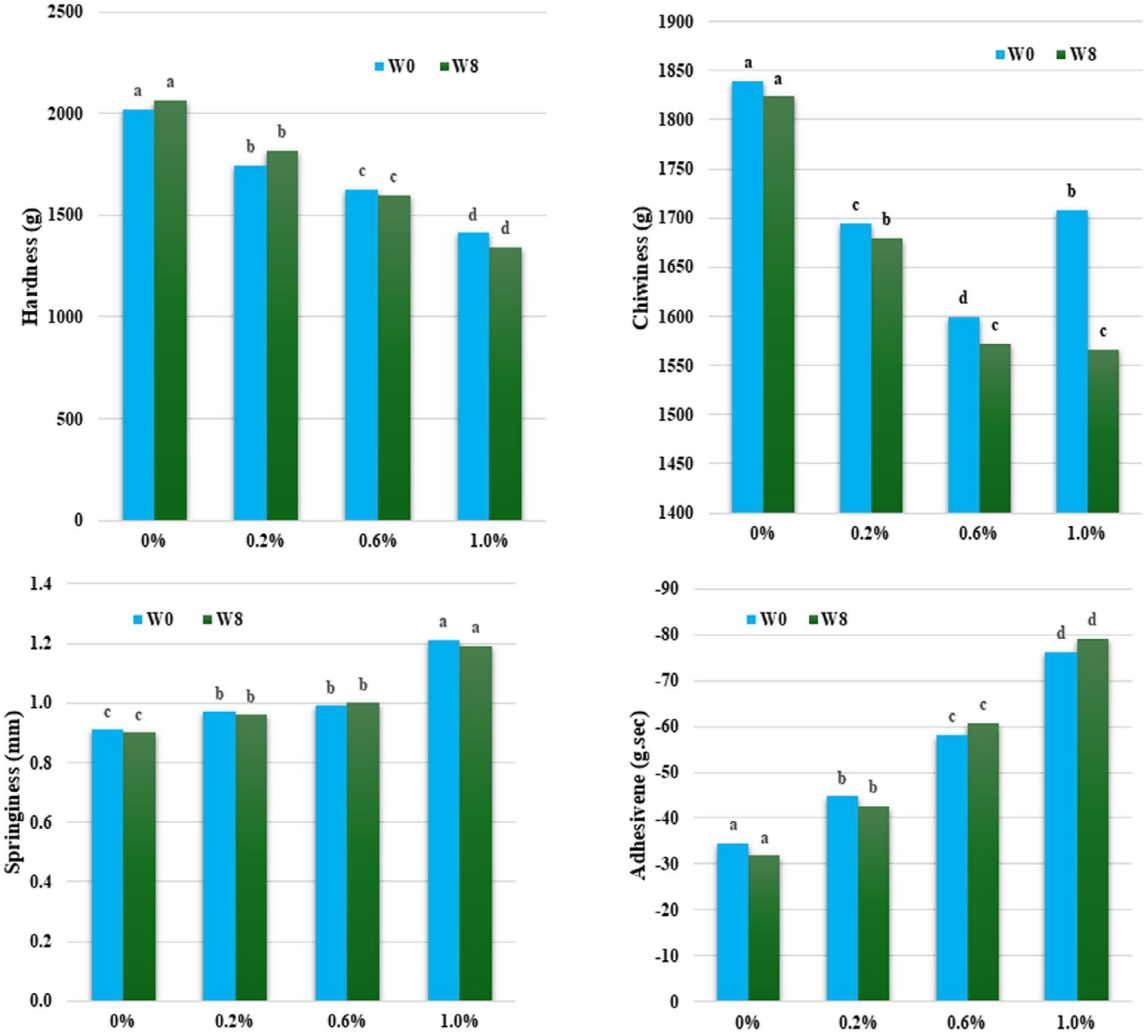
TPA results of CTE fat‐enriched gummy candies after 8 weeks of storage. Different letters above the columns indicate statistically significant differences (*p* < 0.05). Values are presented as mean ± standard deviation (*n* = 3).

Figure 6 illustrates a marked reduction in the hardness of gummy candies with increasing concentrations of CTE. The 1% CTE sample exhibited the lowest hardness value (1341.59 ± 253.69 g), significantly lower than the negative control at 0% CTE (2022.52 ± 112.59 g). This reduction may be attributed to interactions between bioactive compounds in CTE (e. g., anthocyanins, polysaccharides) and the gelatin network, which interfere with the gelation process. Springiness showed a concentration‐dependent increase, with the 1% CTE sample reaching the highest value (1.21 ± 0.03 mm). This suggests that CTE may act as a functional additive enhancing the recovery capacity of the gel matrix after deformation, possibly through flexible interactions with gelatin (Garrido et al. 2015). In contrast, the negative control at 0% CTE exhibited significantly lower springiness values, which remained stable throughout the 8‐week storage period. CTE content also strongly influenced adhesiveness, increasing with higher extract concentrations. The highest adhesiveness was observed in the 1% CTE sample (−79.07 g. sec). This increase may result from the hydrophilic nature of CTE constituents such as anthocyanins, which can enhance surface moisture and increase stickiness (Ozcan et al. 2024). Post‐storage changes in adhesiveness were minimal, indicating surface structure stability under sealed conditions. Chewiness decreased with increasing CTE concentrations, likely due to the disruptive effects of polyphenolic compounds on the gelatin network, reducing elasticity and gel cohesion. The 0.6% CTE sample exhibited the lowest chewiness, while the negative control showed the highest. After 8‐week storage, all samples exhibited a slight reduction in chewiness (< 8.5%). Overall, the observed changes in textural parameters were negligible after 8 weeks of sealed storage. These findings suggest that the gel structure of CTE‐enriched gummy candies remains stable over time. Moreover, CTE contributes to modifying the textural profile toward a softer, more elastic, and flexible consistency, aligning well with consumer sensory preferences.

The gummy candies supplemented with 1.0% CTE exhibited the most optimal textural properties among all tested formulations. It showed the highest springiness, lowest hardness, and the highest yet stable adhesiveness, contributing to a soft, elastic, and pleasant chewing texture. All texture parameters remained stable throughout the 8‐week storage period. Therefore, 1.0% CTE was considered effective in enhancing and maintaining the sensory quality of the gummy product. This concentration was selected for monitoring changes in TAC, TPC, and DPPH antioxidant activity during the 8‐week storage period.

#### 3.5.4 Bioactives During Storage

**FIGURE 7:**
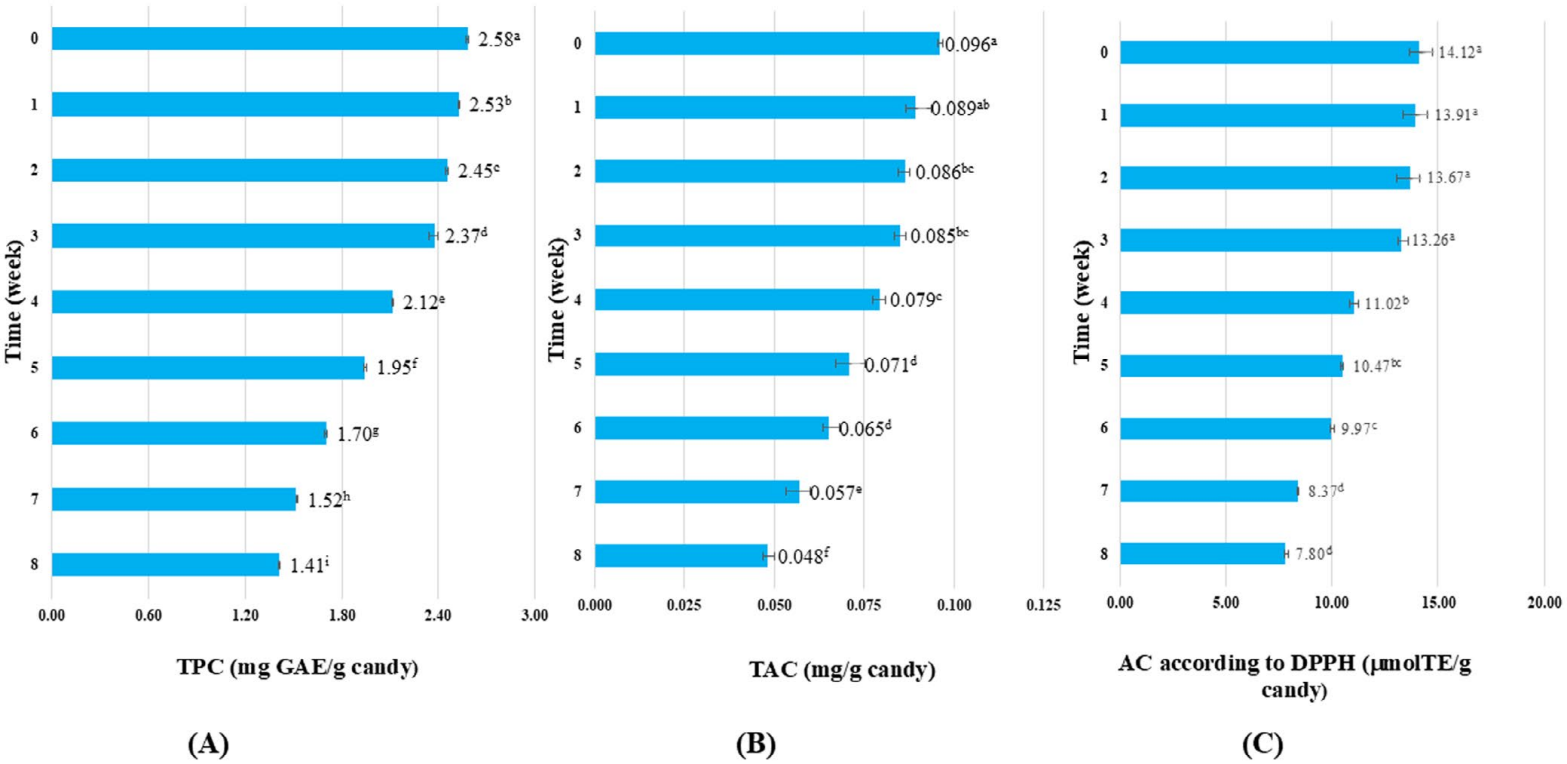
TAC, TPC, and DPPH antioxidant activity results of CTE fat‐enriched gummy candies after 8‐week storage period. Different letters above the columns indicate statistically significant differences (*p* < 0.05). Values are presented as mean ± standard deviation (*n* = 3).

Figure 7 (A, B, C) illustrates the changes in TPC, TAC, and DPPH antioxidant activity of gummy candies supplemented with 1.0% CTE over an 8‐week storage period. The results revealed a gradual decline in all three indicators, with TPC decreasing from 2.58 to 1.41 mg GAE/g candy (~45.3% reduction), TAC dropping from 0.10 to 0.05 mg/g (~50% loss), and DPPH antioxidant activity values falling from 14.12 to 7.80 μmol TE/g (~44.8% reduction). This downward trend is attributed to the degradation of phenolic compounds due to exposure to heat, light, and oxygen, as well as their involvement in free radical scavenging mechanisms. Furthermore, interactions between gelatin and phenolic compounds may limit the bioactivity of these compounds, contributing to the observed decrease in TPC, TAC, and DPPH antioxidant activity values in phenolic‐containing gummy products during storage (Qi et al. 2023). Thus, incorporating 1% CTE enhances the initial bioactive values and contributes to better retention of these properties during long‐term storage.

#### 3.5.5 Lipid Oxidation (PV, TBARS)

**FIGURE 8:**
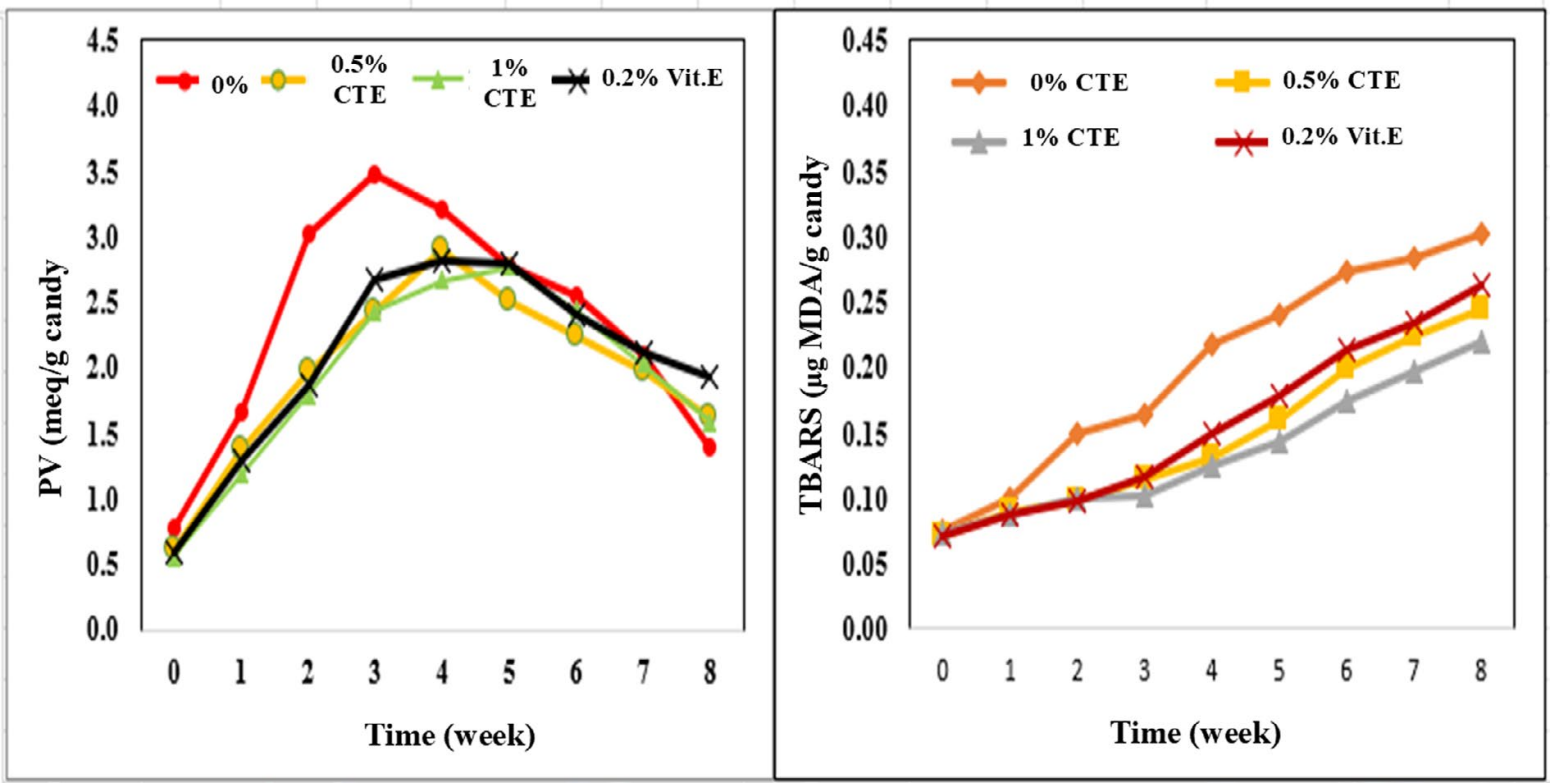
PV, TBARS values of CTE gummy candies after 8 weeks.

As shown in Figure 8, the antioxidant activity of CTE gummy candies was evaluated over an 8‐week storage period using peroxide value (PV) and thiobarbituric acid reactive substances (TBARS) as oxidative markers. The negative control consisted of candies without any antioxidant additives, while the positive control was supplemented with 0.2% (w/w) vitamin E. Experimental samples were formulated with CTE at concentrations of 0%, 0.5%, and 1% (w/w). PV levels increased during the initial storage stage (weeks 0–3 or 4), indicating hydroperoxide formation, and subsequently declined due to the degradation of these primary oxidation products. The negative control exhibited the highest PV, peaking at 3.48 meq/g in week 3, highlighting the pronounced lipid oxidation without antioxidants. In contrast, the 1% CTE sample reached a lower peak PV (2.77 meq/g) at a later time point (week 5), which was even lower than that of the vitamin E‐positive control (2.83 meq/g at week 4), suggesting comparable or enhanced lipid protection relative to vitamin E. Regarding TBARS, all samples exhibited a general increase throughout storage, reflecting the progressive formation of secondary oxidation products such as malondialdehyde (MDA). However, the 1% CTE sample recorded the lowest TBARS value in week 8 (0.2189 μg MDA/g), lower than both the positive control (0.2636 μg MDA/g) and the negative control (0.3018 μg MDA/g). The TBARS inhibition rate reached its highest at week 4 for the 1% CTE sample (42.50%), compared to 31.25% in the positive control, and remained significantly effective at week 8 (27.45% vs. 12.66%). These findings align with previous studies demonstrating that phenolic‐rich plant extracts can delay lipid oxidation by neutralizing free radicals and inhibiting peroxidation chain reactions. Accordingly, CTE exhibited higher antioxidant efficacy than vitamin E and maintained its protective effects over a longer duration, particularly at the 1% concentration. This highlights its potential as a natural alternative to synthetic antioxidants in food preservation (Athukorala et al. 2005; Teets and Were 2008).

Further analysis revealed a gradual decline in total anthocyanin content (TAC), total phenolic content (TPC), and DPPH antioxidant activity throughout the 8‐week storage period at ambient conditions. Among these, anthocyanins degraded most rapidly, retaining only 73.40% of their initial concentration, followed by DPPH (74.48%) and TPC (80.88%). These results confirm that anthocyanins are the primary contributors to antioxidant activity but are also the most susceptible to degradation, primarily through oxidation, glycosidic hydrolysis, and exposure to light and temperature. This degradation trend is consistent with previous reports indicating a~ 20% loss of TAC within 30 days (Abdullah 2008; Vidana Gamage et al. 2021). Additionally, the inclusion of sucrose appeared to stabilize anthocyanins, contributing to extended half‐lives and attenuated losses of TPC and DPPH antioxidant activity. This may also reflect the presence of more stable polyphenolic compounds, such as flavonols, which help sustain antioxidant activity as anthocyanin content declines (Marpaung and Rizki 2020).

Although this study demonstrates its effectiveness in improving oxidative stability of neutral‐pH CTE as a natural colorant and antioxidant in gummy candy, several industrial considerations remain. Compared with other blue colorants such as spirulina phycocyanin and genipin‐based pigments, CTE offers improved color stabilization through acylated anthocyanins and added antioxidant functionality. However, anthocyanins are still sensitive to oxygen, light, and prolonged heating, which may limit stability in some food systems. Regulatory approval, permitted usage levels, and labeling requirements for CTE vary across regions, highlighting the need for compositional standardization and safety compliance. Scalability also requires attention, including extraction yield, solvent recovery, raw material variability, and production cost. While ethanol–water extraction is suitable for green processing, industrial implementation would require process optimization. Finally, anthocyanin performance is matrix‐dependent. Although the gummy system provided a relatively protective environment, stability may differ in beverages, dairy, or high‐temperature processed foods.

The antioxidant performance of CTE in the gummy system is consistent with its compositional characteristics and stability behavior observed throughout the study. Despite the gradual decline in anthocyanin content during storage, the extract maintained effective inhibition of lipid oxidation, as reflected by reduced PV and TBARS values. This indicates that antioxidant activity was not solely dependent on anthocyanins but also supported by co‐existing phenolic compounds, contributing to sustained oxidative protection over time.

Together, these findings highlight the dual functionality of CTE as both a natural colorant and an effective antioxidant, demonstrating its potential for improving oxidative stability in lipid‐containing food systems. Overall, further studies on process scale‐up, regulatory harmonization, and matrix‐specific stabilization strategies are necessary to support broader industrial application.

## 4 Conclusion

This study demonstrated the key influence of pH in extracting and stabilizing anthocyanin compounds from *C. ternatea*, a natural source rich in bioactive constituents. The results indicated that pH 3 was optimal for maximizing TAC, while pH 7 favored the extraction of TPC and DPPH antioxidant activity. UV–Vis spectroscopy, scanning electron microscopy (SEM), and high‐resolution mass spectrometry (LC‐HRMS) revealed significant structural and compositional differences between extracts obtained at pH 3 and pH 7, particularly the presence of acylated anthocyanin derivatives and flavonol glycosides. Extracts obtained at neutral pH (6.8–7.0) demonstrated superior bioactivity and natural color retention when incorporated into gummy candy throughout storage. Notably, at a concentration of 1%, the extract exhibited greater inhibition of lipid oxidation compared to 0.2% vitamin E, as evidenced by significantly lower peroxide value (PV) and thiobarbituric acid reactive substances (TBARS), while offering prolonged lipid protection over time. These findings highlight the potential of *C. ternatea* as a natural colorant with effective antioxidant functionality in lipid‐rich food systems, contributing to the development of clean‐label functional foods based on plant‐derived bioactive compounds.

## Author Contributions

**Thi Anh Dao Dong:** conceptualization, investigation, methodology, writing – review and editing, validation, supervision, formal analysis, writing – original draft. **Ngọc Anh Le:** methodology, software, data curation, formal analysis, investigation. **Thi Thu Tra Tran:** methodology, investigation, data curation, formal analysis, validation. **Kha Duyen Nguyen:** investigation, writing – original draft, methodology, validation, software, formal analysis, data curation, resources, visualization.

## Funding

This research received no external funding.

## Conflicts of Interest

All authors have read and agreed to the published version of the manuscript. The authors declare no conflicts of interest.

## Supporting information


**Table S1:** Preliminary formulation screening based on technical feasibility and basic sensory acceptability for defining the experimental window of CTE and fat incorporation in gummy candy.

## Data Availability

The data that support the findings of this study are available from the corresponding author upon reasonable request.
