# Supplementary material for: Effect of pH on recovery efficiency and anthocyanin composition of Clitoria ternatea extracts with application as lipid antioxidants in food preservation
Source: Food Sci Nutr. 2026 Apr 27;14(5):e71786. doi: 10.1002/fsn3.71786 (PMC13121909; doi:10.1002/fsn3.71786)
Supplement: Supplementary file 1 — Table S1: Preliminary formulation screening based on technical feasibility and basic sensory acceptability for defining the experimental window of CTE and fat incorporation in gummy candy. [file FSN3-14-e71786-s001.docx]

Supplementary Table S1: Preliminary formulation screening based on technical feasibility and basic sensory acceptability for defining the experimental window of CTE and fat incorporation in gummy candy

| **CTE (% w/w)** | **Fat (% w/w)** | **Color acceptability** | **Flavor/Taste acceptability** | **Texture / gel integrity** | **Phase stability** | **Screening decision** |
| --- | --- | --- | --- | --- | --- | --- |
| 0.0 | 0–9 | No characteristic blue color | Neutral | Firm and stable gel | Stable | Control reference |
| 0.5 | 0–6 | Light blue, acceptable | Mild, acceptable | Good gel structure | Stable | Suitable |
| 1.0 | 3–6 | Intense blue, desirable | Balanced, acceptable | Firm and uniform gel | Stable | **Optimal range** |
| 1.5 | 3–9 | Dark blue, slightly excessive at higher levels | Slight astringency at higher concentration | Slightly softer gel structure | Stable to slightly unstable at high fat | Upper acceptable limit |
| 2.0 | 6–9 | Overly dark | Noticeable astringency | Weak gel structure | Partial phase separation | Not selected |
| 3.0 | 9–12 | Excessively dark | Unacceptable (strong astringency) | Poor gel formation due to interference with gelatin network | Clear phase separation observed | Excluded |
